# Diversity Analysis in *Cannabis sativa* Based on Large-Scale Development of Expressed Sequence Tag-Derived Simple Sequence Repeat Markers

**DOI:** 10.1371/journal.pone.0110638

**Published:** 2014-10-20

**Authors:** Chunsheng Gao, Pengfei Xin, Chaohua Cheng, Qing Tang, Ping Chen, Changbiao Wang, Gonggu Zang, Lining Zhao

**Affiliations:** 1 Key Laboratory of the Biology and Process of Bast Fiber Crops, Ministry of Agriculture, Changsha, China; 2 Institute of Bast Fiber Crops and Center of Southern Economic Crops, Chinese Academy of Agricultural Sciences, Changsha, China; 3 Biotechnology Research Center, Shanxi Academy of Agricultural Sciences, Taiyuan, China; Washington State University, United States of America

## Abstract

*Cannabis sativa* L. is an important economic plant for the production of food, fiber, oils, and intoxicants. However, lack of sufficient simple sequence repeat (SSR) markers has limited the development of cannabis genetic research. Here, large-scale development of expressed sequence tag simple sequence repeat (EST-SSR) markers was performed to obtain more informative genetic markers, and to assess genetic diversity in cannabis (*Cannabis sativa* L.). Based on the cannabis transcriptome, 4,577 SSRs were identified from 3,624 ESTs. From there, a total of 3,442 complementary primer pairs were designed as SSR markers. Among these markers, trinucleotide repeat motifs (50.99%) were the most abundant, followed by hexanucleotide (25.13%), dinucleotide (16.34%), tetranucloetide (3.8%), and pentanucleotide (3.74%) repeat motifs, respectively. The AAG/CTT trinucleotide repeat (17.96%) was the most abundant motif detected in the SSRs. One hundred and seventeen EST-SSR markers were randomly selected to evaluate primer quality in 24 cannabis varieties. Among these 117 markers, 108 (92.31%) were successfully amplified and 87 (74.36%) were polymorphic. Forty-five polymorphic primer pairs were selected to evaluate genetic diversity and relatedness among the 115 cannabis genotypes. The results showed that 115 varieties could be divided into 4 groups primarily based on geography: Northern China, Europe, Central China, and Southern China. Moreover, the coefficient of similarity when comparing cannabis from Northern China with the European group cannabis was higher than that when comparing with cannabis from the other two groups, owing to a similar climate. This study outlines the first large-scale development of SSR markers for cannabis. These data may serve as a foundation for the development of genetic linkage, quantitative trait loci mapping, and marker-assisted breeding of cannabis.

## Introduction

*Cannabis sativa* L., a member of the Cannabinaceae, is a diploid (2n = 20) monocotyledon, and one of the oldest cultivated plants. It has been cultivated or grows wild around the world, and is used in diverse applications [1]. Cannabis is a botanical genus of flowering plants that is divided into two species. Hemp, which has low tetrahydrocannabinol (THC) content, is mainly used to cultivate for fiber and seed production, whereas marijuana, with its relatively higher THC content, is used for its psychoactive potency [2]. Although cannabis cultivation is being restricted in many countries due to its potential use as a recreational drug, there has been a resurgence of interest in cannabis for its agronomic potential. In fact, hemp was used for textile production in China more than 6,000 years ago, and is still widely grown in China now.

Identification of variability among its genotypes is the key factor in any crop's improvement. Molecular markers play an important role in selecting these diverse genotypes. During the initial stages of development of molecular markers, some types of markers were used to analyse the genetic diversity in cannabis. These included random amplified polymorphic DNA (RAPD) [3]–[5], amplified fragment length polymorphisms (AFLP) [6]–[8], and inter simple sequence repeat amplification (ISSR) [9]. However, these markers have the common shortcoming of poor repeatability and dominance. Single nucleotide polymorphism (SNP) was mainly used for studies of specific genes in cannabis [10]. More recently, microsatellite markers were proven highly useful in applied breeding programs [11]. Microsatellites, known as simple sequence repeats (SSRs), are tandem repeats of short (1 to 6 bp) DNA sequences that exist throughout the entire genome of eukaryotic organisms, including both non-coding and coding regions. SSRs are among the most useful genetic markers in biology. SSR loci have many distinguishing features, such as high information content, co-dominant inheritance patterns, consistent distribution along chromosomes, reproducibility, and locus specificity [12]–[14]. Furthermore, SSRs demonstrate an impressive ability to be transferred among related species, making them excellent markers for comparative genetic and genomic analyses [15]. Therefore, SSRs are widely used for genetic studies in recent years. However, only small-scale SSR markers have been reported in cannabis [1], [2], [16], which is far from sufficient for effective genetic mapping and marker-assisted breeding. The complete genome and transcriptome sequence of cannabis was published in 2011 [17], providing a foundation for cannabis research. Nowadays, identification of SSRs from expressed sequence tags (ESTs) is the preferred method for rapid and inexpensive marker development. Furthermore, these EST-SSRs can then be directly linked to the genes that confer important agronomic traits. Thus, SSRs based on the ESTs have become one of the most important methods in agriscience for the analysis of genetic diversity, genotype identification, high density genetic mapping, molecular tagging of genes, and marker assisted selection (MAS) breeding [18]. There are many reports about the application of EST-SSR markers in various plants at present, including sugar cane [19], barley [20], grapes [21], Chinese cabbage [22], and citrus [23]. Therefore, developing EST-SSR in cannabis on a large scale is important and urgent.

In the present study, potential SSR loci were characterized from the cannabis ESTs [17] and large-scale development of EST-SSRs in the genus was performed for the first time. This was followed with evaluation of the quality of the novel SSR markers and subsequent selection of a group of them for genetic diversity analysis. These results will provide a valuable resource for genetic and genomic studies of cannabis for genetic studies.

## Materials and Methods

### Plant materials

One hundred and fifteen cannabis varieties were used for the polymorphic analysis of SSR markers. Among them, 100 varieties were from 18 provinces of China, Longdama 1 (LDM), Jinma 1 (JM1), Wandama 1 (WDM), Yunma 1 (YM1), Yunma 4 (YM4), Yunma 5 (YM5) and Yunwan 6 (YW6) were cultivated varieties and the others were all wild varieties in China. The other 15 varieties were from Ukraine, Poland, and France, respectively (Table S1). Seeds for the study were collected from the Institute of Bast Fiber Crops, China Academy of Agriculture Science, Changsha, China.

### DNA extraction and quantification

The cannabis seeds were grown in pots under natural conditions. DNA was extracted from seedlings (100 mg) by the cetyltrimethyl ammonium bromide (CTAB) method [24]. After extraction, 3 µL of the DNA sample (for all varieties) was loaded in 1.0% agarose gels. A DNA marker was loaded as a control to assess the quality and the quantity of DNA. Based on the marker standards, DNA samples were normalized to a uniform concentration (approximately 10 ng/µL) and used for SSR genotyping.

### SSRs sources and primer development

Potential SSR markers were detected among the 32,324 sequences using the AutoSSR software [17], [25]. The parameters were adjusted for identification of perfect di-, tri-, tetra-, penta-, and hexanucleotide motifs with a minimum of 9, 6, 5, 4, and 3 repeats, respectively. The ESTs sequences were used to design primers flanking the potential SSRs. Input criteria for the Primer 3.0 software for designing primers were as follows: length, 17–23 bp; GC content, 40–60%; and estimated amplicon size, 100–400 bp [26]. Primers were synthesized by Sangon Biotech (Shanghai) Co., Ltd.

### SSRs amplification and detection of polymorphisms

Approximately, 10 ng of template DNA was added to a 10-µL PCR mix containing 1×PCR buffer with MgCl_2_ (Perkin-Elmer), 250 µM dNTPs, 0.2 µM primers (forward and reverse), and 0.5 units of TaqGold DNA Polymerase (Perkin-Elmer). The PCR reaction profile comprised a 10-min incubation at 94°C, then a cycle of 94°C for 30 s, 50–60°C for 30 s and 72°C for 40 s, repeated 35 times. Following cycling, the reaction was held at 72°C for 10 min, before a final 4°C hold. The entire reaction is carried out in the BioRAD S1000 Thermal cycler. The PCR product quality was checked in a 1.0% agarose gel, using 3 µL of the PCR reaction and the remaining reaction was then subjected to electrophoresis on a 8% polyacrylamide gel, consisting of 30% PA (acrylamide+N, N-methylene bisacrylamide, Biosharp) 12 mL, 10×TBE 4 mL, TEMED 50 µL, 10% APS (ammonium peroxydisulfate, Biosharp) 950 µL, and ddH_2_O to a total volume of 45 mL. Electrophoresis was carried out in 1×TBE buffer at 220 U for 90 min. Gels were stained with 0.1% silver nitrate following a chromogenic reaction with 1.5% NaOH (including 1% formaldehyde), and finally photographed in white light. We screened 24 typical cannabis varieties to assess the usefulness of the SSR primer pairs developed in this study.

### Determination of genetic diversity among 115 cannabis varieties

Forty-five markers (Table S2) were selected from the 117 EST-SSR primers developed to analyse the relationship of cannabis varieties. The allelic data were converted into a binary matrix using the score 1, 0 for presence and absence of the allele. The binary data were analysed using the Numerical Taxonomy Multivariate Analysis System (NTSYS-pc) version 2.10 software [27]. Genetic similarity (GS) coefficients were calculated based on the coefficient for similarity matching by using the SIMQUAL module of the software. Using the GS matrix, we constructed a dendrogram using the unweighted pair group method with arithmetic average (UPGMA) to determine genetic relationships among the 115 genotypes. Principal Coordinate Analysis (PCoA) was also performed using NTSYS-pc software to resolve the patterns of clustering among genotypes. The effective allele number (Ne), Shannon's informative index (I), expected heterozygosity (HE), and the percentage of polymorphic loci (PP) were calculated by Popgen Ver.132 [28].

## Results

### Development and characterization of SSR markers

In this study, EST-SSR loci were detected from the 32,324 EST sequences of the cannabis transcriptome using AutoSSR software [17], [25]. A total of 4,577 potential SSR loci were identified from 3,624 EST sequences (Table 1). The frequency of occurrence for SSRs was 1 SSR per 8.49 kb, and 11.21% of the EST sequences contained SSR loci. Among the 3,624 EST sequences, 505 sequences contained 2 SSR loci, 114 sequences contained 3 SSR loci, 33 sequences contained 4 SSR loci, and 16 sequences contained more than 4 SSR loci. Moreover, 121 sequences contained several SSR motifs that were present in a compound formation. Finally, a total of 3,442 SSR markers were developed and made publicly available (Table 1, Table S3).

**Table 1:** Results of searches for EST-SSRs in cannabis.

Search item	Numbers
Total number of sequences searched	32324
Total size of sequences searched (bp)	39038131
Total number of SSRs identified	4577
Total number of SSR-containing sequences	3624
Number of sequences containing more than 1 SSR	668
Dinucleotides	748
Trinucleotides	2334
Tetranucleotides	174
Pentanucleotides	171
Hexanucleotides	1150
Marker number of SSRs present in compound formation	121
Total number of SSR markers developed	3442

In order to further characterize the potential SSR loci, the types, frequencies, and distributions of the 4,577 SSRs were analysed. The result indicated that trinucleotide was the most abundant (2,334, 50.99%) type of repeat. The number of hexanucleotide, dinucleotide, tetranucleotide, and pentanucleotide markers were 1150 (25.13%), 748 (16.34%), 174 (3.8%), and 171 (3.74%), respectively (Table 1). Table 2 presents the lengths of the EST-SSRs based on the number of repeat units. The results showed that 3,385 SSRs (73.96%) were calculated to range from 18 to 22 bp. Only 90 SSRs (1.97%) were longer than 40 bp (Table 2).

**Table 2:** Frequency of EST-SSRs in cannabis.

Motiflength	Repeat numbers								
	3	4	5	6	7	8	9	10	≥11
Di							265	142	341
Tri				1262	557	249	158	51	57
Tetra			126	42	6				
Penta		137	25	9					
Hexa	931	177	30	8	3	1			

**Figure 1:**
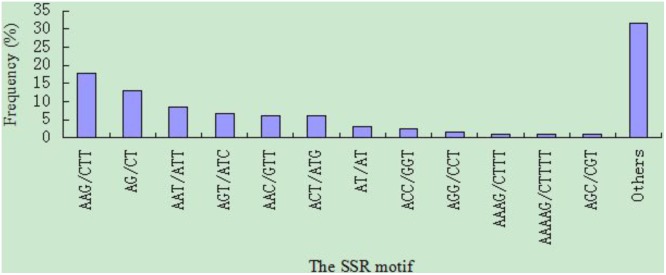
Frequency distribution of cannabis EST-SSR based on motif numbers.

Among the 3,442 EST-SSRs a total of 318 motif sequence types were identified. The abundance of each motif was 3, 10, 19, 51, and 235 of dinucleotide, trinucleotide, tetranucleotide, pentanucleotide, and hexanucleotide repeats, respectively. The most abundant type of repeat motif was the AAG/CTT trinucleotide repeat (822, 17.96%), followed by AG/CT (590, 12.89%), AAT/ATT (395, 8.63%), AGT/ATC (303, 6.62%), AAC/GTT (278, 6.07%), ACT/ATG(276, 6.03%), AT/AT (141, 3.08%), ACC/GGT (111, 2.43%), AGG/CCT (68, 1.49%), AAAG/CTTT (52, 1.13%), AAAAG/CTTTT (50, 1.09%), and AGC/CGT (47, 1.03%), respectively (Figure 1). The other 306 motifs (1444, 31.55%) not specifically mentioned were also presented to be entered into the database in addition to the above repeats motifs (Figure 1).

One hundred and seventeen primer pairs were randomly selected to evaluate the quality of the SSR markers across 24 cannabis varieties (Table S1). Amplified DNA fragments were observed with 108 markers, while 9 markers failed to generate amplicons. A total of 119 loci were successfully amplified by the 108 SSR markers. Among the 24 cannabis varieties tested, 21 loci showed no polymorphism. Among the polymorphic loci, 62, 24, 8, 1, 2, and 1 each had 2, 3, 4, 5, 6, and 7 alleles, respectively.

### Evaluation of genetic relationships among cannabis varieties

Based on these results, 45 high-quality primer pairs (56 loci) were selected to evaluate the genetic diversity and relatedness of the 115 cannabis varieties listed in Table S1. The number of alleles per locus ranged from 2 to 7 among the 115 varieties, with an average of 2.87. Considering 2 varieties as a variety pair, 6555 variety pairs were found among the 115 varieties. Among 6555 variety pairs, the largest polymorphic ratio (99.38%) was observed between 53 and 54 and the two varieties were all from Shanxi1 province. The smallest polymorphic ratio (31.01%) was observed between 107 and Y5, 107 was from Europe and Y5 was from Yunnan province (Table S4).

**Figure 2:**
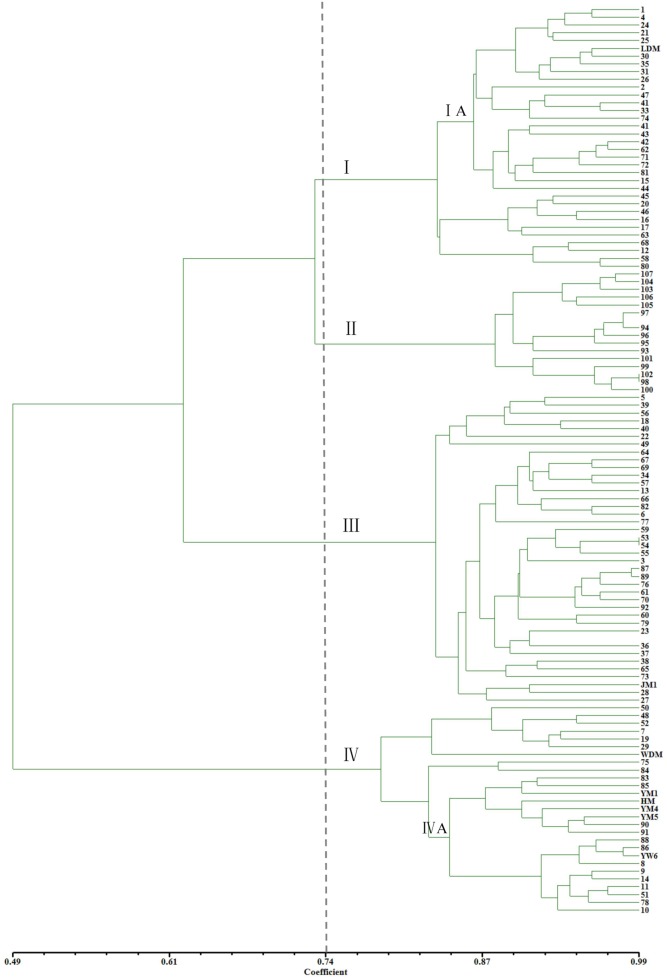
Dendrogram derived using UPGMA cluster analysis based on the SSR genetic similarity coefficient for 115 cannabis varieties.

The genetic similarity (GS) coefficients between the cannabis varieties were calculated in the SIMQUAL module of NTSYS-pc [28]. Using a GS score of 0.74 as the threshold, the 115 cannabis varieties could be classified into 4 clusters (Figure 2). The results showed that 34 Northern China (Heilongjiang, Liaoning, Jilin, Neimenggu, and Xinjiang provinces) varieties, 27 Southern China (Anhui, Chongqing, Jiangsu, Zhejiang, Yunnan and Guangxi provinces) varieties, and 39 Central China (Ningxia, Hebei, Henan, Gansu, Shandong, Shanxi1, and Shanxi2 province) varieties fell into clusters I, IV and III, respectively. Fifteen varieties originated from Europe (Ukraine, Poland, and France), and were assigned as individual cluster II.

**Figure 3:**
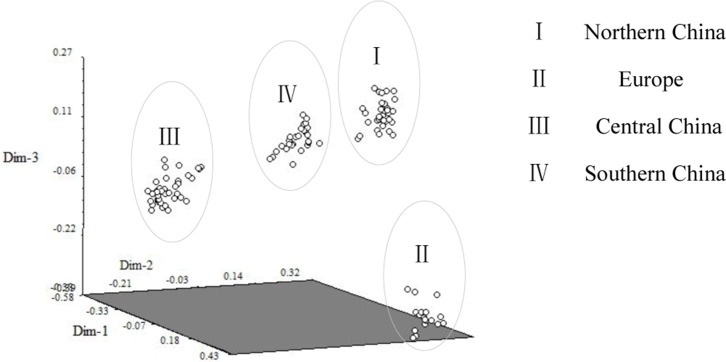
Principles coordinate analysis for SSR markers using the genetic similarity matrix for 115 cannabis varieties.

In PCoA, the three main axes explained approximately 62% of the total variation, at 36.4%, 16.8%, and 8.7%, respectively. The 115 cannabis varieties could also be distinctly classified into 4 groups,which was consistent with the results of cluster analysis (Figure 3).

Based on the above results, 115 cannabis varieties could be divided into 4 groups mainly according to the geographical distribution: Northern China, Europe, Central China and Southern China. Then the effective allele number (Ne), Shannon's informative index (I), expected heterozygosity (HE), and the percentage of polymorphic loci (PP) values were calculated for the 4 groups. The results showed that the highest level of genetic diversity was observed in the Central China group. Data in descending order, the Shannon's informative index was III, IV, I, II; the expected heterozygosity was III, IV, I, II; the percentage of polymorphic loci was III, I, IV, II (Table 3).

**Table 3:** Variability parameters in 4 groups of cannabis.

Cluster	Origin	Number ofvarieties	Ne	I	HE	PP (%)
I	Northern China	34	1.7747	0.5518	0.3548	76.36
II	Europe	15	1.3423	0.2776	0.1820	41.82
III	Central China	39	1.8008	0.5800	0.3698	85.45
IV	Southern China	27	1.7743	0.5602	0.3565	74.55

## Discussion

### Development and characterization of EST-SSR markers of cannabis

Simple sequence repeats (SSRs) are important molecular markers for genetic mapping and variety identification in cannabis and other plants. However, lack of sufficient SSR markers has limited the development of cannabis molecular genetics. In this study, 3,442 EST-SSR markers were identified for the first time. The frequency of EST-contained SSRs was 11.21%. This frequency was higher than the reported fiber crops flax (3.5%) and ramie (3.83%) [29], [30], but lower than other reported plants such as coffee (18.5%) and oilseed rape (15.58%) [31], [32]. The difference was also reflected in the average distance between SSRs, which was 8.49 kb in cannabis, but 16.5 kb and 19.3 kb in flax and ramie [29], [30]. In addition, two of the most abundant motifs were trinucleotide and hexanucleotide repeats in cannabis, while dinucleotide and trinucleotide repeats were the most abundant motifs in flax and ramie. Interestingly, the EST-SSR distribution in cannabis was similar to that in melons [33]. A previous study had revealed that AG/CT was the most abundant class of the isolated SSRs, representing 50% overall in cannabis [2]. However, our data demonstrated that AAG/CTT was the most abundant motif (17.96%). The discrepancy may result from the different techniques utilized in developing the SSR markers. Probe technology was used to develop markers in the previous study, while this study had a transcriptome database (via AutoSSR software) at its disposal. The frequency of the motif here was similar to other dicots, including citrus and melon [23], [33]. Gao hypothesized that AAG/CTT was an advantageous repeat motif in dicots, and that its higher frequency may be related to the increased use of the corresponding proteins [34]. As there is no doubt that cannabis is a dicot, these data suggest that the results presented here may be closer to the actual frequency of the motif.

In order to evaluate the quality of the primers designed, 117 SSRs were randomly selected to amplify their target sequences among 24 cannabis varieties. Of these, 108 (92.31%) SSRs successfully yielded amplicons. Given that the SSRs were randomly selected, the amplification efficiency may be considered to be the actual availability of all 3,442 identified SSRs. Nine SSRs failed to generate amplicons, most likely due to the location of the primer across splice sites, the presence of large introns, and association with poor-quality sequences [20], [29]. In addition, 5 amplicons deviated from their expected sizes. This phenomenon was also noted in other species, and may be due to introns or large insertions, among other reasons [35], [36]. All 115 varietals selected for diversity testing came from the hemp species, while the ESTs came from *Cannabis sativa* Purple Kush, a marijuana strain that is widely used for its medicinal effects [17]. Species differences between marijuana and hemp may therefore have affected the amplification efficiency. Nevertheless, the observed 92.31% amplification efficiency indicated that the developed EST-SSRs were of high quality, and may serve as a good foundation for future research of cannabis.

### Genetic diversity and climate influences of cannabis

Selection and use of genetically diverse genotypes are key factors in any crop breeding program in order to develop cultivars with a broad genetic base. Hemp is a dioecious annual that commences its reproductive cycle when photoperiods are shorter than a critical length [37], such that day length and temperature may determine the floral transition and flowing times [38], [39]. One of the most important traits for hemp is the timing of the transition from a vegetative to flowering state, which can control the growth period, as well as affecting the fiber quality and yield. For example, Northern European hemp grown in Southern Europe performs poorly due to premature flowering, resulting in shorter vegetative periods that limit stem growth, whereas the opposite occurs when Southern European varieties are grown in Northern Europe [38], [40]. Similar phenomena were also found in China. Climate zones are defined as areas with distinct climates, and are classified according to the average and the typical ranges of different variables such as temperature and precipitation. Sunshine intensity and day length in different latitudes are the main factors affecting temperature. Therefore, climate zones are closely related to latitude. Our results showed that 100 hemp varieties from China could be classified into 3 distinct clusters, and that the 3 clusters were consistent with the cool temperate, warm temperate, and subtropical zones in China, respectively. These indicated that the climate, created by latitude, temperature, and day length, is a key factor affecting the germplasm diversity of hemp. Although the three clusters from China were physically closer, hemp in Northern China had a greater similarity coefficient with European hemp than with the other two clusters. This is most likely because these two regions are at similar latitudes, and thus have similar climatic conditions. The varieties from Heilongjiang and Yunnan provinces were individually classified in clusters IA and IVA (Figure 2), perhaps owing to the higher latitude (i.e. the colder environment) and low latitude plateaus, respectively. Our results provide a new insight into the study on germplasm resources and systematic classification in hemp, which may be helpful for the introduction, germplasm development, and utilization in different climates, countries, or continents.

In conclusion, this is the first report on large-scale development of SSR markers in cannabis and provides guidance for germplasm introduction and utilization. Future studies with these SSR markers could be useful for conservation programs, identification activities, and breeding procedures.

## Supporting Information

Table S1 **Description of 115 cannabis varieties used in this study.**
(DOC)

Table S2 **One hundred and seventeen EST-SSR markers used for PCR amplification.**
(XLS)

Table S3 **The information of 3443 EST-SSR markers developed.**
(XLS)

Table S4 **The polymorphic ratios among 115 cannabis varieties.**
(XLS)
