# Supplementary material for: Diversity Analysis in Cannabis sativa Based on Large-Scale Development of Expressed Sequence Tag-Derived Simple Sequence Repeat Markers
Source: PLoS One. 2014 Oct 20;9(10):e110638. doi: 10.1371/journal.pone.0110638 (PMC4203809; doi:10.1371/journal.pone.0110638)
Supplement: Table S1 — Description of 115 cannabis varieties used in this study. (DOC) [file pone.0110638.s001.doc]

**Table S1** Description of 115 cannabis varieties used in this study

| code | origin | cluster | code | origin | cluster |
| --- | --- | --- | --- | --- | --- |
| LDM* | Heilongjiang | Ⅰ | 35 | Gannan, Heilongjiang | Ⅰ |
| JM1* | Shanxi1 | Ⅲ | 36 | Zhidan, Shanxi2 | Ⅲ |
| WDM* | Anhui | Ⅳ | 37 | Huanglong, Shanxi2 | Ⅲ |
| YM1* | Yunnan | Ⅳ | 38 | Fuxian, Shanxi2 | Ⅲ |
| YM4* | Yunnan | Ⅳ | 39 | Yangyuan, Hebei | Ⅲ |
| YM5* | Yunnan | Ⅳ | 40 | Xinyang, Henan | Ⅲ |
| YW6 | Yunnan | Ⅳ | 41 | Baiquan, Heilongjiang | Ⅰ |
| HM | Guangxi (Huoma) | Ⅳ | 42 | Sunwu, Heilongjiang | Ⅰ |
| 1 | Daqing, Heilongjiang | Ⅰ | 43 | Linjiang, Jinlin | Ⅰ |
| 2 | Jilin | Ⅰ | 44 | Fuyu, Jinlin | Ⅰ |
| 3 | Fenyang, Shanxi1 | Ⅲ | 45 | Yushu, Jinlin | Ⅰ |
| 4 | Zhaozhou, Heilongjiang | Ⅰ | 46 | Zhaoan, Jinlin | Ⅰ |
| 5 | Ningxia | Ⅲ | 47 | Changtu, Liaoning | Ⅰ |
| 6 | Liangshan, Shandong | Ⅲ | 48 | Yeji, Anhui | Ⅳ |
| 7 | Shuyang, Jiangsu | Ⅳ | 49 | Jingyuan, Ningxia | Ⅲ |
| 8 | Xiangyun, Yunnan | Ⅳ | 50 | Chongqing | Ⅳ |
| 9 | Chuxiong, Yunnan | Ⅳ | 51 | Yongren, Yunnan | Ⅳ |
| 10 | Dayao, Yunnan | Ⅳ | 52 | Anhui (Hanma) | Ⅳ |
| 11 | Zhenkang, Yunnan | Ⅳ | 53* | Shanxi1 (Huangzhangma) | Ⅲ |
| 12 | Qitai, Xinjiang | Ⅰ | 54 | Shuoxian, Shanxi1 | Ⅲ |
| 13 | Lvxian, Shandong | Ⅲ | 55 | Guangling, Shanxi1 | Ⅲ |
| 14 | Cangyuan, Yunnan | Ⅳ | 56 | Henan (Xianma) | Ⅲ |
| 15 | Tonghua, Jinlin | Ⅰ | 57 | Cangshan, Shandong | Ⅲ |
| 16 | Moqi, Neimenggu | Ⅰ | 58 | Huocheng, Xinjiang | Ⅰ |
| 17 | Tuoketuo, Neimenggu | Ⅰ | 59 | Pingyi, Shandong | Ⅲ |
| 18 | Wuzhong, Ningxia | Ⅲ | 60 | Tancheng, Shandong | Ⅲ |
| 19 | Suining, Jiangsu | Ⅳ | 61 | Dunhuang, Gansu | Ⅲ |
| 20 | Xifeng, Liaoning | Ⅰ | 62* | Tonghe, Heilongjiang | Ⅰ |
| 21 | Nenjiang, Heilongjiang | Ⅰ | 63 | Neimenggu | Ⅰ |
| 22 | Jiuquan, Gansu | Ⅲ | 64 | Xishan, Henan | Ⅲ |
| 23 | Zhouqu, Gansu | Ⅲ | 65 | Shenmu, Shanxi2 | Ⅲ |
| 24* | Weihe, Heilongjiang | Ⅰ | 66 | Luanchuan, Henan | Ⅲ |
| 25* | Wuchang, Heilongjiang | Ⅰ | 67 | Bijiang, Henan | Ⅲ |
| 26 | Beian, Heilongjiang | Ⅰ | 68 | Jiageda, Neimenggu | Ⅰ |
| 27 | Dingxiang, Shanxi1 | Ⅲ | 69 | Fangcheng, Henan | Ⅲ |
| 28 | Shuoxian, Shanxi1 | Ⅲ | 70 | Kangxian, Gansu | Ⅲ |
| 29 | Xianqiao, Zhejiang | Ⅳ | 71 | Heihe, Heilongjiang | Ⅰ |
| 30 | Wangkui, Heilongjiang | Ⅰ | 72 | Nehe, Heilongjiang | Ⅰ |
| 31 | Qinggang, Heilongjiang | Ⅰ | 73 | Zhidan, Shanxi2 | Ⅲ |
| 32 | Dayangshu, Neimenggu | Ⅰ | 74 | Zhangwu, Liaoning | Ⅰ |
| 33 | Arong, Neimenggu | Ⅰ | 75 | Tongxiang, Zhejiang | Ⅳ |
| 34 | Laiwu, Shandong | Ⅲ | 76 | Longxi, Gansu | Ⅲ |
| 77 | Xiangcheng, Henan | Ⅲ | 93* | Glukhov 18, Ukraine | Ⅱ |
| 78 | Yunlong, Yunnan | Ⅳ | 94* | Zolotonosha 15, Ukraine | Ⅱ |
| 79 | lvxian, Shandong | Ⅲ | 95* | Ukraine  (Dniprovs'ki Odnodomni 6) | Ⅱ |
| 80 | Maqi, Xinjiang | Ⅰ |
| 81 | Keshan, Heilongjiang | Ⅰ | 96 | Ukraine (Dniprovs'ki 14) | Ⅱ |
| 82 | Xiangcheng, Henan | Ⅲ | 97 | Ukraine (YUSO-14) | Ⅱ |
| 83 | Tongxiang, Zhejiang | Ⅳ | 98* | Ukraine (K-176) | Ⅱ |
| 84 | Bama, Guangxi | Ⅳ | 99* | Poland (Beniko) | Ⅱ |
| 85* | Bama, Guangxi | Ⅳ | 100* | Poland (Dzikipolski) | Ⅱ |
| 86* | Dali, Yunnan | Ⅳ | 101 | Poland (Bialobrzeskie) | Ⅱ |
| 87* | Fenyang, Shanxi1 | Ⅲ | 102 | Poland (Dolnoslaskie) | Ⅱ |
| 88 | Kunming, Yunnan | Ⅳ | 103* | France (Fedora 17) | Ⅱ |
| 89 | Linxian, Shanxi1 | Ⅲ | 104* | France (Epsilon 68) | Ⅱ |
| 90* | Yunnan (YM460) | Ⅳ | 105* | France (Felina 32) | Ⅱ |
| 91* | Yunnan (YM535) | Ⅳ | 106 | Ukraine (USO-31) | Ⅱ |
| 92 | Yangqu, Shanxi1 | Ⅲ | 107 | France (Futura) | Ⅱ |

*cannabis varieties used to evaluate the quality of the EST-SSR markers
